# Endoreduplication underlies body size variation in monomorphic ants

**DOI:** 10.1186/s12983-026-00619-0

**Published:** 2026-06-15

**Authors:** Mateusz Okrutniak, Olga Jabłońska, Alicja Frost, Grzegorz Nowak, Adam Świerczyński, Irena M. Grześ

**Affiliations:** 1 Department of Zoology and Animal Welfare, University of Agriculture in Kraków, al. Mickiewicza 24/28, Kraków, 30‑059 Poland; 2 Department of Zoology, University of Warmia and Mazury in Olsztyn, ul. Oczapowskiego 1 A, Olsztyn, 10‑719 Poland

**Keywords:** Endoreduplication, Body size, Flow cytometry, *Lasius* spp.

## Abstract

**Background:**

In ants, morphological variation is particularly important, as it constitutes a key mechanism underlying the division of labor. Despite this, relatively few studies have addressed the proximate cellular mechanisms responsible for worker size variation. Endoreduplication represents a promising contributor to morphological diversity, as it is a process with a well-established cellular basis.

**Methods:**

In this study, we investigated whether endoreduplication can account for variation in body size in monomorphic ants, analogous to patterns previously reported in polymorphic species. We tested whether body size correlates with the level of endoreduplication in workers of three monomorphic ant species that differ in ecology, colony structure, and life-history traits. The analysis showed that individual worker size was positively correlated with nuclear DNA content (C-value), both in the overall linear mixed-effects model (LMM) and in species-specific analyses. As an additional finding, the C-value level exhibited body-part specificity, reaching the highest values in the abdomen.

**Conclusion:**

Our results demonstrate that endoreduplication contributes to body size variation not only in ants with distinct worker subcastes, but also in species with subtle size variability. This suggests that endoreduplication represents an evolutionarily ancient and potentially widespread mechanism, which may have served as a developmental foundation for the evolution of advanced polymorphism in ants.

## Background

Although social insects represent only a small fraction of global insect diversity, they dominate nearly every terrestrial ecosystem, often reaching extraordinary population densities. Many traits set these insects apart, yet perhaps the most striking hallmark of social life is the ability of colonies to perform many different tasks simultaneously, in the same place and at the same time. In this sense, the colony functions as a *superorganism*, exhibiting a mode of life radically different from that of solitary insects [57, 5, 21]. This contrast becomes especially clear when considering the limitations faced by solitary species: engaging in one vital activity inevitably prevents another. For example, food cannot occur while securing shelter or avoiding predators.

Eusocial insects have overcome such constraints through the division of labor among colony members. Different groups of workers can forage, defend, and construct simultaneously, often side by side. Much of the empirical and theoretical foundation on division of labor comes from one of the most prominent groups of eusocial insects: the ants. With over 15,700 described species and subspecies, virtually all ants are social, with the exception of a few parasitic taxa [42]. Importantly, in ants task allocation usually is not fixed: an individual may switch roles depending on its age, size, or morphology, as well as in response to the immediate needs of the colony [16, 39, 50].

The ability of ant colonies to perform multiple tasks simultaneously arises from the high degree of variability among workers. This variability spans several levels, including genetic, neurobiochemical, age-related, behavioral, and morphological differences. Genetic diversity resulting, for example, from multiple paternity can lead to patrilines differing in task propensity [34, 45]. Neurobiochemical and sensory variation, encompassing differences in pheromone sensitivity and neurotransmitter levels, shapes individual responsiveness to colony cues and thereby improves the efficiency of task distribution [18]. Age-related polyethism drives the temporal sequence of tasks performed during a worker’s lifetime, while behavioral variation generates inter-individual differences in exploration, risk-taking, or responsiveness to signals [43]. Finally, morphological polymorphism, manifested in distinct worker castes, enables physical specialization for tasks such as defense or brood care [29, 25]. Taken together, this multidimensional variability ensures both division of labor and the flexibility required for the colony to function as a superorganism.

In some ant species, workers are divided into distinct subcastes, such as minors, majors (often referred to as soldiers), and in some cases supermajors or supersoldiers. These species are described as polymorphic. The worker size-frequency distributions in polymorphic ants can exhibit pronounced multimodality; however, in many species the distribution is closer to continuous, with a subset of distinctly larger workers still recognized as a separate subcaste [21]. Among polymorphic species, the most complex subcaste systems - those including supermajors - are restricted to only five genera: *Atta*, *Daceton*, *Eciton*, *Pheidologeton*, and *Pheidole* [22]. The evolutionary significance of such a complex caste system lies in the specialization of workers for performing unique functions within the colony. Soldiers are usually specialized for defense or other limited tasks, and unlike minor workers, they do not undergo age-related changes in task performance. For example, in African driver ants, long-mandibled soldiers are assigned to hunt large prey and defend nestmates during group foraging, whereas large-headed majors of *Pheidole*, with strong adductor muscles, open seeds [21]. In leaf-cutter ants, the smallest workers in the colony (called minims) ride on leaf fragments carried back to the nest by larger workers, defending foragers against parasitoids, particularly parasitoid flies [52]. Although ant polymorphism can enhance division of labor, it is important to note that 75% of ant genera are monomorphic [21]. In contrast to polymorphic species, monomorphic species lack distinct worker subcastes. Their workers show a symmetrical, unimodal size distribution, with relatively low variation in body size.

The distinction between monomorphic and polymorphic ant species serves as a practical framework for comparing species that differ in worker caste organization, ranging from relatively simple systems to more complex and derived forms of worker polymorphism.

Yet this dichotomy, while convenient, can misleadingly suggest a profound biological divide between monomorphic and polymorphic species when in fact the two share many similarities. First, monomorphism does not mean uniformity. Some monomorphic species harbor consistent, species-specific variation in worker size. In *Formica fusca*, the relative difference between the worker with the smallest and the largest head size is approximately 44% [7]. Secondly, monomorphism does not preclude a link between worker body size and the tasks they perform. Even subtle size differences can underlie meaningful divisions of labor within the colony. Pioneering work by Herbers and Cunningham [19] first demonstrated that, in the monomorphic ant *Leptothorax longispinosus*, the largest workers were those most likely to forage. Since then, a growing body of research has revealed that size-based division of labor is not an exception but a recurring feature across other monomorphic ants [17, 33, 51, 54]. In *Formica*, for instance, workers differing subtly in body size are channeled into distinct non-reproductive roles such as honeydew collection, nest construction, or food gathering. The tightness of this link, however, varies across species and tends to weaken in taxa where worker size ranges are narrower [53].

The unusual morphology of polymorphic ants naturally raises the question: what factors shape this variation? A range of multiple factors, such as developmental and evolutionary limits, larval diet and social interactions have been proposed to explain worker size variation in polymorphic ants (e.g., [22, 55]; reviewed in [58]). However, while these explanations emphasize ultimate ecological and evolutionary factors, relatively few studies have considered proximate cellular mechanisms that might underlie size variation. One candidate mechanism is endoreduplication. Endoreduplication is a modified cell cycle in which DNA replication occurs without subsequent mitosis or cytokinesis (reviewed in [26, 60]; reviewed in [38]). As a result, the cell bypasses division and accumulates multiple copies of its genome. The consequence of this process is polyploidy (endopolyploidization), characterized by the presence of multiple genome copies within a single cell which may lead to the formation of enlarged nuclei. The increased gene copy number enhances the cell’s transcriptional and translational capacity, thereby supporting elevated metabolic activity, protein synthesis, and storage capacity (reviewed in [31]). These functional advantages are particularly evident in secretory or rapidly proliferating tissues, positioning endoreduplication as an efficient, low-cost evolutionary strategy to enhance cellular performance without the need for additional cell proliferation [14].

Intensive endoreduplication at the organismal level may contribute to an increase in body size. This relationship arises from the fundamental observation that elevated DNA content is associated with an expansion of nuclear volume, which may subsequently lead to an increase in overall cell volume ([35]; reviewed in: [11, 14, 31]). This relationship is commonly described by the concept of the “karyoplasmic ratio” [8, 20]. Because organismal growth can proceed via two distinct pathways, either through an increase in cell number or through an increase in cell size, it is reasonable to hypothesize that endoreduplication influences body size primarily by modulating cell volume within tissues. Empirical support for this hypothesis has been provided by studies on the nematode *Caenorhabditis elegans* [15, 27], a species characterized by eutely, i.e., a constant number of somatic cells. In this model organism, experimental manipulation of endoreduplication levels resulted in corresponding changes in adult body size, with increased endoreduplication leading to larger individuals and reduced levels producing smaller ones. Despite these findings, the cellular mechanisms underlying the relationship between endoreduplication and organismal body size remain insufficiently understood. Furthermore, some studies suggest that an increase in cell volume may precede the accumulation of additional DNA, implying that endoreduplication could be a consequence rather than a primary driver of changes in cell size [24]. Taken together, current evidence indicates that increased endoreduplication has the potential to influence organismal body size; however, this relationship is context-dependent and not universally deterministic. The potential consequences of endoreduplication have led myrmecologists to question its role in the functioning of ant societies. Recent findings suggest that, although endoreduplication is not a direct determinant of caste, it is a contributor to inter-caste variation in physiology and morphology [40, 41, 48, 49]. In a study of four polymorphic species, Scholes et al. [41] found that larger workers exhibit higher levels of endopolyploidy than smaller ones. This work has been particularly inspiring for our research, as it points to endoreduplication as a possible driver of morphological variation within the worker caste. Furthermore, endoreduplication levels in ants have been shown to differ markedly among body segments [41, 49], reaching the highest values in abdomen and comparable values in thorax and head. Scholes at al. [40] demonstrated that organs with high metabolic demand, such as the Malpighian tubules in the abdomen, show the greatest degree of endoreduplication, whereas nervous tissue, which dominates the head, is characterized by comparatively low levels.

Building on these findings, we asked whether endoreduplication, as a mechanism increasing body size variation in polymorphic ants, might also operate in species traditionally regarded as monomorphic. If supported, this would indicate that endoreduplication represents a common mechanism generally across ants, contributing to body size variation within the worker caste. This would further imply that endoreduplication represents a starting point for the generation of phenotypic variation, which may contribute to the emergence of division of labor and ultimately to advanced forms of worker polymorphism and size-based task specialization, hallmark features of ants as social insects. However, as our study did not include behavioural analyses, we do not directly assess the relationship between endoreduplication and division of labor. Instead, our aim was solely to determine whether the level of endoreduplication correlates with body size. The study was conducted on three species of the genus *Lasius*, *Lasius flavus*, *Lasius fuliginosus*, and *Lasius niger*, which differ in their biology, thereby enabling broader inference in the case of consistent results. We conducted supplementary analyses of endoreduplication across the head, thorax, and abdomen of *L. niger* workers. *Lasius niger* is a well-established model species in myrmecological studies. These measurements were based on a relatively small dataset - a single species and a single colony and were intended to verify whether ploidy levels measured across different body segments align with patterns previously reported in the literature, with the highest endoreduplication observed in the abdomen and relatively low, comparable levels in the head and thorax.

## Materials and methods

### Study species

This study was conducted on three monomorphic *Lasius* ant species: the yellow meadow ant (*Lasius flavus*), the jet ant (*Lasius fuliginosus*) and the black garden ant (*Lasius niger*). These species were selected because they are common in the vicinity of our laboratory, are not legally protected, and their workers can be readily collected without the use of destructive or invasive methods. Moreover, each species can be easily identified in the field, and their nests contain numerous workers. The proximity of these species to the laboratory enabled the analysis of fresh (non-frozen) material, which was required by the methodology used for measuring cellular ploidy.

*Lasius flavus* is a moderately thermophilous and ubiquist species [10]. It inhabits open, sun-exposed habitats, including meadows, pastures, and urban lawns. Nests are constructed below ground and often form small mounds covered with herbs. The species is predominantly subterranean and opens its nests only during nuptial flights [6, 10, 46]. The body length of workers ranges from about 2.2 to 4.8 mm [36].

*Lasius fuliginosus* is a moderately thermophilous oligotopic species associated primarily with deciduous forests, but also occurring in mixed and coniferous stands. It is a dendrobiont that nests in cavities beneath the trunks and roots of usually living trees. The body length of workers ranges from about 4 to 5 mm [10, 36].

*Lasius niger* is a eurytopic species exhibiting high ecological tolerance and biological plasticity. Colonies typically occur in open, sun-exposed, and moderately dry habitats such as meadows, pastures, and urban green areas [10, 32, 44]. The body length of workers ranges from about 3.5 to 5 mm [36].

### Experimental design and replication

For each species, workers were collected in the field from three independent colonies originating from the same field site. Approximately 60 workers were sampled per colony. For each individual worker, two measurements were obtained: endoreduplication level (C-value) and body size (head width, HW). Each worker was treated as a biological replicate. Colony identity nested within species was included as a random effect in linear mixed-effects models to account for non-independence among workers originating from the same colony.

### Collection of ant workers

Ants used in this study were collected in northern Poland (Olsztyn region). Workers of *L. niger* and *L. flavus* were collected from an abandoned university garden in Jaroty (53.730937, 20.475909), whereas *L. fuliginosus* was sampled from lime trees forming a roadside alley near Kaplityny (53.805615, 20.629249). Workers of all species were collected using a handheld vacuum aspirator. *L. niger* and *L. flavus* were sampled from three independent ground nests per species (nest diameter: 30–40 cm; minimum distance between nests: ≥5 m), whereas *L. fuliginosus* workers were collected from three trees located approximately 200 m apart. For analyses of intra-body ploidy variation, *L. niger* workers were obtained from a single nest. Each nest sample comprised approximately 150 workers, except for the intra-body ploidy analysis, for which approximately 25 workers were collected. Immediately after collection, ants were transported to the laboratory and subjected to flow cytometric analysis within 24 h of sampling. Individuals used to investigate the relationship between endopolyploidy and body size were sampled in June 2025, whereas workers used to assess intra-body ploidy variation were obtained in September 2025.

From each of the nine nest samples (three species × three nests), 50 workers were randomly selected for morphological analysis. The body size of each worker was measured as the maximum head width above the eyes (“HW” according to [10]). Head width is a commonly used estimator of the body size of ants, including *Lasius* ants [2, 12, 59]. Measurements were taken under 100× magnification using a Nikon SMZ 1270 stereomicroscope equipped with a digital camera. Head widths were measured to the nearest 0.00001 mm from digital images using NIS-Elements BR software. All measurements were conducted in the Laboratory of the Department of Zoology, University of Warmia and Mazury. In total, 450 individuals were measured. Measurements were performed in a series of five individuals. Each worker was euthanized with ethyl acetate immediately prior to measurement, and subsequently subjected to ploidy analysis. The time between euthanasia and ploidy measurement was approximately 20 min.

### Flow cytometric analysis

Ploidy level determination by flow cytometry was conducted following the procedure described in Jablonska et al. [23] and Duda et al. [13], using the ready-to use CyStain UV Precise T kit (Sysmex Partec GmbH, Görlitz, Germany) with slight modifications. Briefly, at the beginning of the experiment, whole ant tissue was used to investigate the relationship between endopolyploidy and body size. Fresh tissue from each individual of the three analyzed ant species (50 individuals per species) was placed in a small Petri dish containing 0.3 ml of nuclear extraction buffer and mechanically chopped using two scalpels. The resulting suspension was transferred to 1.5 ml Eppendorf tubes and incubated at room temperature for 5 min, with occasional gentle mixing by finger vortexing. The suspension was then filtered through a 30 µm nylon mesh filter (CellTrics, Sysmex Partec GmbH, Gorlitz, Germany) into cytometric tubes. After that, 0.7 ml of staining buffer containing 4’,6-diamidino-2-phenylindole (DAPI) was added through the filter to the nuclei suspension, which was then incubated for 3 min prior to measurement.

Flow cytometric analyses were performed using CyFlow Cube 6 Ploidy Analyzer (Sysmex Partec GmbH, Görlitz, Germany). Each measurement began by adjusting the instrument gain to position the fluorescence peak of the standard (a previously verified haploid ant) at a mean channel value of approximately 100. Subsequently, unknown samples were analyzed individually. For each sample, at least 400 nuclei were measured at a flow rate below 100 nuclei s^− 1^ to determine ploidy levels. Nuclear population gating and quantification of nuclei at each ploidy level were performed using CyFlow software integrated with the CyFlow Cube 6 flow cytometer. To investigate intra-body ploidy variation, following the same procedure as for whole individuals, ploidy levels were analyzed separately in the head, thorax, and abdomen of *L. niger* workers (20 individuals) to assess intra-body ploidy variation. Endopolyploidy level was expressed as a C-value. This metric was derived from the number of nuclei detected at each ploidy level for each individual ant or dissected body part. The C-value was calculated following the formula proposed for female ants by Scholes et al. [41]:

$$\begin{aligned}\mathrm{C-value}=&(0\,\cdot\,n_{2c}+1\,\cdot\,n_{4c}+2\,\cdot\,n_{8c})/\\&(n_{2c}+n_{4c}+n_{8c}),\end{aligned}$$

where *n*_2*c*_, *n*_4*c*_, *n*_8*c*_ - number of nuclei at each ploidy level

### Statistical analysis

The effects of body size (head width, HW) and species on C-value were analysed using two complementary linear mixed-effects modelling approaches. First, we fitted a model including all three species, with HW, species, and their interaction as fixed effects, and colony identity (nested within species) included as a random intercept. Colony was treated as a random effect to account for non-independence of individuals originating from the same colony and to capture colony-level variation not explained by the fixed effects. We consider testing the interaction between species identity and HW to evaluate whether the relationship is consistent across species or species-dependent. We did not have strong a priori predictions regarding which species might exhibit a stronger or weaker relationship, because there is a lack of studies linking endoreduplication to differences in ant biology that could provide a basis for such predictions.

Second, we fitted species-specific models for each of the three species separately to examine within-species relationships between HW and C-value. Analyses were conducted in Python using the *statsmodels.smf.mixedlm* package with a unique variable identifying the species-colony combination used as the grouping factor. Accordance with the normal distribution was checked based on the residual distribution.

Differences in C-value among the head, thorax, and abdomen were initially analyzed using a parametric test. As the residuals did not meet the assumption of normality, we used the non-parametric Kruskal-Wallis test to compare medians. The Kruskal-Wallis test was performed using the *scipy.stats* package in Python (version 3.12.7).

## Results

To evaluate whether endoreduplication contributes to variation in worker body size in monomorphic ants, we quantified C-values using flow cytometry in three species - *Lasius niger*, *Lasius flavus*, and *Lasius fuliginosus* - and analysed their relationship with body size both across species (using a linear mixed-effects model) and within species (using species-specific models). We additionally examined, using workers of *L. niger*, whether endoreduplication is segment-specific, with the expectation that it would be highest in the abdomen.

### Flow cytometry measurements

Flow cytometry measurements were obtained from 443 individual workers representing nine colonies (45–50 individuals per colony). In the worker bodies of the three studied species, both diploid (2 C) and tetraploid (4 C) cells were present. In *L. fuliginosus*, however, octaploid (8 C) cells were additionally detected. The occurrence of these octaploid cells in *L. fuliginosus* resulted in higher C-values compared with the other species.

### Single linear mixed-effects model (LMM) including all three species

**Fig. 1:**
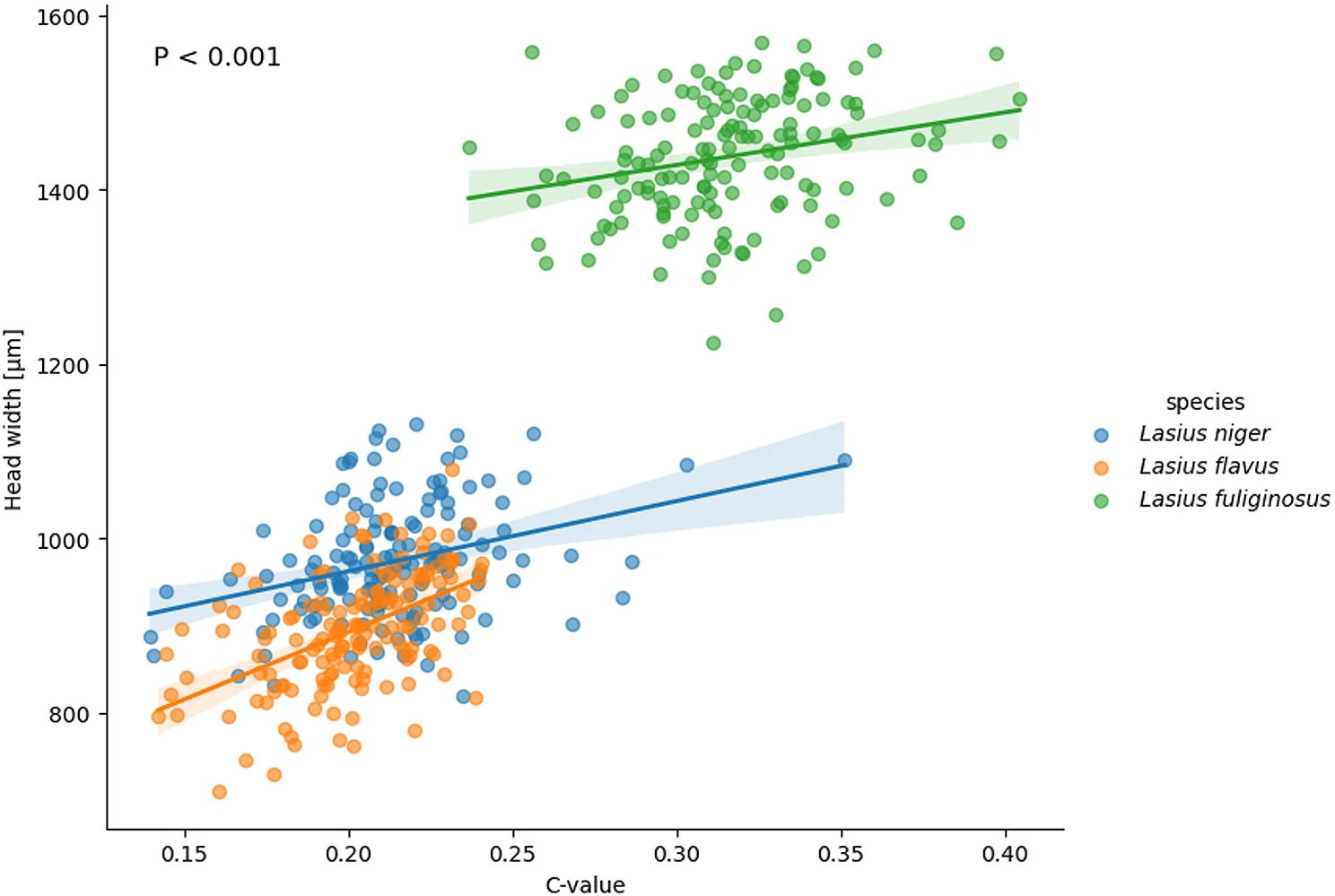
Result of the combined linear mixed-effects model (LMM) including all three species showing the relationship between head width and C-value across the studied species. Head width serves as an estimator of individual worker body size. The C-value represents the relative proportion of somatic cells with ploidy levels higher than 2C. Each dot represents a single worker

In the combined linear mixed-effects model including all three species, C-value increased significantly with body size (head width, HW; z = 5.33, *P* < 0.001, Fig. 1). Species identity had no significant effect on C-value (*L. fuliginosus*: z = 1.01, *P* = 0.314; *L. niger*: z = − 0.11, *P* = 0.909; *L. flavus* used as the reference species). The HW × species interaction was not significant for either species (*L. fuliginosus*: z = − 0.35, *P* = 0.729; *L. niger*: z = 0.12, *P* = 0.903), indicating similar scaling relationships between body size and C-value across species. Variation attributable to colony identity was negligible. To verify model assumptions, we examined standard diagnostic plots, including residuals versus fitted values and quantile–quantile (Q-Q) plots. These diagnostics indicated no substantial violations of homoscedasticity or normality. Inspection of standardized residuals revealed a small number of potential outliers (6 of 443 observations; ~1.4%). Exclusion of these observations did not alter the statistical significance of the tested effects, supporting the robustness of the results.

### Species-specific models fitted separately for each of the three species

In all three species (*L. niger*,* L. flavus* and *L. fuliginosus*), C-value increased significantly with head width (HW; mixed-effects models, all *P* < 0.001). Intercepts differed among species but were always significantly greater than zero or marginally so. Variance explained by colony identity was negligible in all models, indicating that the relationship between body size and C-value was consistent across colonies within species.

### Differences in C-value among the head, thorax, and abdomen

**Fig. 2:**
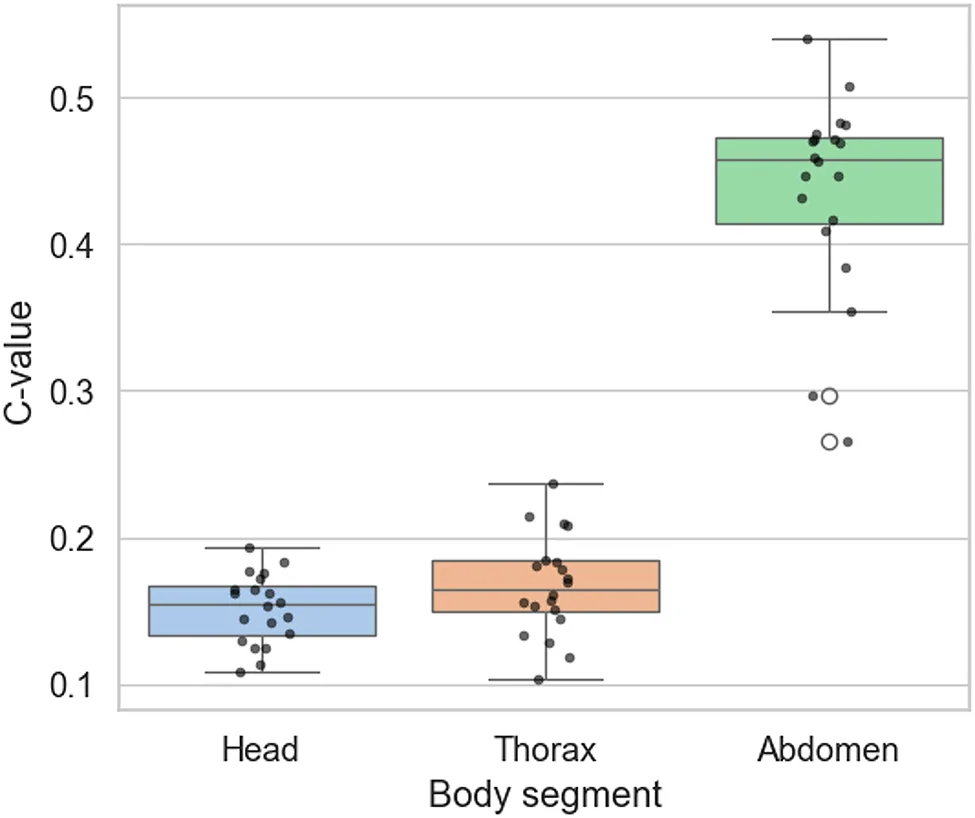
Boxplot showing the results of the Kruskal-Wallis test comparing C-values among body segments of *Lasius niger* workers. Boxes represent interquartile ranges (IQR), horizontal lines indicate medians, and whiskers show the data range excluding outliers. Individual observations are shown as filled dots, while outliers are additionally marked with adjacent open circles

Kruskal-Wallis test revealed significant differences among segments (H = 40.122, *P* < 0.001; Fig. 2). Post-hoc pairwise comparisons using Dunn’s test with Holm correction showed that the median C-value was significantly higher in the abdomen compared to both the head and thorax, whereas no significant difference was detected between head and thorax (*P* > 0.05). These results, while not central to the main experimental hypothesis, complement the primary analyses by confirming that the C-value is segment-specific, with the highest values observed in the abdomen.

## Discussion

The ability of social insects to perform multiple tasks simultaneously arises from multidimensional variability among workers, encompassing genetic, physiological, neurobiochemical, behavioral, and most notably morphological differences. In recent years, there has been growing interest in uncovering the cellular mechanisms underpinning body-size variation in ants. These studies have highlighted endoreduplication as a promising cellular mechanism potentially underlying body-size variation [41, 48, 49].

In parallel, some studies have gone one level deeper, focusing on genetic determinants of worker size variation [1, 37]. For instance, quantitative methylation of *Egfr* has been shown to explain a substantial proportion of body-size variance in *Camponotus floridanus*. Given that *Egfr* is a key regulator of cell-cycle progression and growth signalling, and that endoreduplication is closely tied to cell-cycle regulation, these findings suggest a link between *Egfr* signalling and endoreduplication levels, which are in turn associated with variation in worker body size in this species [1, 41].

Evidence linking endoreduplication to worker body size in ants has so far been derived from studies of polymorphic species, which represent a relatively narrow subset of ants, leaving open questions about its generality. We demonstrated that across the three monomorphic ant species, endopolyploidy levels (C-value) increased significantly with worker head width (HW), indicating a consistent positive association between cell DNA content and body size. In consequence, we suggest that endoreduplication is more widespread in ants than previously appreciated, extending also to monomorphic species exhibiting markedly low variance in body size. Because monomorphic ants are generally considered to represent an evolutionarily older condition relative to polymorphic species [22, 4], this broader distribution implies that endoreduplication is likely an evolutionarily ancient feature of ant societies. Taken together, our findings lead us to propose that endoreduplication was already present early in ant evolution. By generating subtle morphological variation among workers, endoreduplication may therefore have provided a foundation for simple, size-based division of labor, as observed today in some monomorphic ant species.

Endoreduplication is genetically encoded, but its expression is strongly modulated by developmental and environmental factors ([3]; reviewed in [31, 47]). This combination of genetic control and environmental plasticity allows markedly different phenotypes to arise from the same genotype, making endoreduplication both heritable and responsive to ecological conditions. As a result, endoreduplication may not only contribute to body-size variation but also function as a compensatory mechanism buffering the consequences of low genetic diversity. This is particularly relevant in ant colonies, where high relatedness among workers, especially in species headed by a single, singly mated queen, represents a strong constraint on the production of morphological and functional diversity. Under such conditions, ants face strong evolutionary pressure to generate phenotypic variation through mechanisms that are not strictly genotype-dependent. Endoreduplication provides a plausible means to fulfill this role, enabling phenotypic variation even in genetically uniform colonies. Although our study focuses on ants, similar genotype-phenotype decoupling mediated by endoreduplication may occur in other insects and animal taxa, where this mechanism could likewise increase morphological diversity without a corresponding increase in genetic diversity.

Although all three study species belong to the genus *Lasius*, they differ substantially in ecology, colony structure, and life-history traits. *Lasius flavus* represents a strictly subterranean lifestyle, *L. niger* is a surface-foraging ecological generalist, and *L. fuliginosus* is a highly social, partly arboreal species forming large, structurally complex colonies. This range of ecological and social strategies highlights that the observed consistency in the relationship between body size and endoreduplication is unlikely to be idiosyncratic to a single species. Nevertheless, broader phylogenetic sampling, including, for example, *Formica*, *Myrmica*, *Leptothorax*, *Temnothorax*, will be essential to test whether this mechanism is conserved across monomorphic ants more generally.

Demonstrating that endoreduplication shapes body size in both mono- and polymorphic ants does not imply that it is the sole mechanism underlying body size variation in ants. In ants, variation in body size is more likely to arise from the combined effects of multiple interacting factors. In polymorphic ants, worker subcastes emerge through divergence in larval developmental trajectories, driven by the canalization of gene expression towards specific caste phenotypes [58]. These alternative developmental pathways are triggered by changes in nutritional input and hormonal regulation, mediated by nutrient-sensitive signaling pathways such as the IIS/TOR/insulin-like systems [56, 9, 28, 30]. Together, these factors modulate transcriptional programs and growth rates, resulting in pronounced differences in worker size and morphology (e.g., soldiers versus minor workers). In contrast, monomorphic ants lack discrete worker subcastes, and worker larvae follow broadly similar developmental pathways. Nevertheless, the trajectory of individual larvae - and consequently the body size of adult workers - can still be modified by environmental factors similar to those shaping body size variation in polymorphic ants [55]. In consequence, endoreduplication does not generate worker castes on its own, but it may broaden the phenotypic variance expressed along an already defined developmental axis [41].

Previous studies have shown that in ant workers, polyploid cells are not randomly distributed across the body [40, 41, 49]. When worker tagmata (head, thorax, abdomen) were analysed separately by flow cytometry, the highest levels of endoreduplication were consistently observed in the abdomen, whereas levels in the head and thorax were comparable. Subsequent dissection-based studies helped to explain these differences [40]. The abdomen contains organs and tissues with high metabolic activity, in which endoreduplication levels are particularly elevated. Thus, endoreduplication in ants does not occur randomly but shows clear organ- and tissue-specific patterns. In the present study, the highest level of endoreduplication was also found in the abdomen. This result supports the notion of tissue-specific endoreduplication, suggesting that this pattern may be conserved across ant lineages. However, as the analysis of endoreduplication across tagmata was secondary to the main objective of the study (i.e. the relationship between endoreduplication and body size), the limited sample size (*N* = 20, single colony) precludes broader mechanistic or population-level inferences. Taking into account the results of our study, as well as previously cited work, we can therefore suggest that elevated ploidy in ant workers may not primarily function to generate variation in body size per se, but may instead be more closely associated with the metabolic performance of tissues involved in key physiological processes. In such a scenario, any association between increased body size and endoreduplication could arise indirectly through enhanced tissue function. Improved tissue performance could, in turn, affect overall organismal growth and development, potentially contributing to differences in body size. However, this interpretation remains speculative and requires further empirical testing. Future studies with larger sample sizes and multi-colony designs would be required to explore potential mechanistic links between endoreduplication patterns and tissue function.

## Conclusions

Our results provide evidence for a relationship between the level of endoreduplication and body size. We show that in three monomorphic ant species, endoreduplication levels are positively correlated with worker body size, despite clear differences in their biology. This indicates that endoreduplication contributes to generating body-size variation in monomorphic ants, paralleling patterns previously documented in polymorphic species. A key question for future research is how endoreduplication shapes functional and behavioural variation - not only in ants, but across other social insects. Taken together, these results suggest that endoreduplication is an evolutionarily conserved mechanism that helps generate subtle worker-size variation and may have facilitated the later evolution of more elaborate worker polymorphisms in ants.

## Data Availability

All data supporting the findings of this study, including morphometric measurements, cytometric analyses and the Python script used to reproduce the statistical analyses are available in the Zenodo repository: https://zenodo.org/records/19894924.
